# Opinion: The use of point-of-care sonography in primary care: An ethical perspective

**DOI:** 10.4102/safp.v64i1.5410

**Published:** 2022-01-11

**Authors:** Shane D. Murphy

**Affiliations:** 1 Department of Family Medicine, Faculty of Health Sciences, University of the Witwatersrand, Johannesburg, South Africa

**Keywords:** point of care ultrasound, capacity-building, education, primary care, healthcare delivery

## Abstract

The use of point-of-care ultrasound (PoCUS) is fast becoming a global standard of care. World over, training programmes are embracing the use of PoCUS to improve patient care, reduce risks to patients and reduce the burden of unnecessary referrals and investigations. The South African setting is particularly amenable to the benefits of this diagnostic modality to improve healthcare delivery to rural and marginalised populations. However, as with any technological advancements, the use of PoCUS is also not immune to deleterious consequences and patient harm from overdependence and over-zealous uptake. Family physicians, as the champions of primary care, should take the lead to ensure the effective and proper use of PoCUS in rural and primary care settings through advocacy, training and accreditation of clinicians to safely harness the benefits of PoCUS and minimise harm.

## Introduction

Point-of-care ultrasound (PoCUS) is the use of ultrasonic devices to produce real-time images by the attending physician as part of the primary diagnostic assessment and for therapeutic guidance.^1^ The interest and use of PoCUS are on the rise globally, increasingly considered a standard of care. A systematic review of 26 meta-analyses and 168 primary clinical studies^2^ found high sensitivity and specificity of ultrasound in the primary care setting, often superior to other diagnostic imaging modalities. Ultrasound has been shown to reduce diagnostic delay and supplement clinical management decisions, and is regarded by the World Health Organization (WHO) as an essential diagnostic imaging modality in primary care.^3^

In primary care, PoCUS has been shown to increase the physician’s ability to detect heart failure accurately, improving management. It was also more sensitive in detecting pneumonia compared to chest x-ray and clinical examination combined.^2^ In patients presenting with dyspnoea, for example, PoCUS detected pathological diagnoses in 14% of the patients that had been missed on the physician’s history and examination alone.

Further, experienced PoCUS users were 100% accurate at diagnosing abdominal aortic aneurysm – comparable to the accuracy amongst radiologists.^2^ Point-of-care ultrasound for the detection of deep vein thrombosis showed a sensitivity of 96% and specificity of 97%.^1^ Similarly, the use of PoCUS has been validated for several other applications in primary care, including the detection of biliary pathology (cholecystitis and cholelithiasis), hydronephrosis/nephrolithiasis, lung pathology, soft tissue injuries and infections and musculoskeletal injuries.

The appeal of PoCUS has raised credible concerns around its uptake, with critics citing several pitfalls^4,5^ around unregulated use with consequent medical negligence and maleficence. The relative novelty of PoCUS allows limited evaluative casuistry to inform current practice. However, current literature^2,3^ shows that general practitioners, trained in its use, can safely apply ultrasound in a wide range of clinical settings markedly improving diagnostic accuracy, enhancing management decisions, lowering healthcare costs and reducing exposure to ionising radiation.

## Uptake

Point-of-care ultrasound allows for real-time, dynamic images that facilitate the direct correlation of symptoms with signs. Emergency medicine has shown the fastest uptake of PoCUS with 96%, 95%, 89% and 82% of emergency departments in the United States (US), rural Canada, Denmark and South Korea, respectively, having ultrasound available.^1^ Furthermore, training in the use of PoCUS is increasingly used in emergency medicine training programmes, with some countries holding it as a mandatory requirement for fellowship with their respective college (including South Africa).^6^

Comparatively, primary care has been slow to absorb this practice, with an average global uptake of less than 3.0%; and data for usage in South Africa are unknown.^3^ A systematic review of family medicine programmes in developed nations found that 2.2% of programmes had fully-established PoCUS training programmes, whilst 40.0% of universities had either recently started a programme or were planning to do so.^3^ Consistent with this trend, the American Academy of Family Physicians passed a resolution to include PoCUS as part of their training a decade ago in 2011.^3^

A central premise of this movement is that PoCUS must only be used to supplement clinical assessment and decision-making, and not replace clinical acumen and evidence-based practice. Translating this to everyday practice, PoCUS should only be used along the latter stages of hypothetico-deductive reasoning to complement clinical reasoning and evidence-based practice. For example, in the patient who presents with unilateral lower limb oedema, clinical reasoning should facilitate sufficient pre-test probability^3^ to safely use ultrasound to either support or refute the potential diagnosis of deep vein thrombosis.

Despite the evidence suggesting that the general practitioners trained in the use of PoCUS can safely use ultrasound in a wide range of clinical settings to guide diagnosis and management, family physicians have not embraced this technology to improve patient care. The College of Family Physicians of South Africa (CFPSA) holds that their duties and functions are to maintain and advance the standard of family medicine, identify ethical issues and set and maintain standards, particularly in postgraduate education.^7^

Currently, the CFPSA provides no discussion or consideration of the use of PoCUS. This is an omission by the council because several of the core standards in the family medicine postgraduate programme can be linked to PoCUS.^7^ For example:

- Core Standard 1: Vision of high-quality, evidence-based care.
- Core Standard 2: Evaluate and manage patients with undifferentiated problems cost-effectively.
- Core Standard 3: Facilitate the health of the family and community.

Next, we will review the ethical considerations in adopting this (relatively) new technology.

## Beneficence

The Health Professions Council of South Africa^8^ (HPCSA) describes the ‘duty’ of healthcare professionals to promote good practice, provide beneficial treatment and to be fair and just. Healthcare professionals must be concerned about the best interests of the patient and promote access to care.^8^ Furthermore, the HPCSA claims that practitioners have a duty to improve and maintain their own performance by engaging in ongoing educational activities.

Moodley et al.^9^ noted that beneficence is not limited to the level of the individual, but rather that the international community has a collective ‘duty of care’ to ensure that adequate and affordable measures are available to those most in need. Other important dimensions of beneficence include clinical competence and risk-benefit analysis.

The onus is on the practitioner to continuously develop knowledge and skills with a commitment to life-long learning. Evidence-based medicine drives clinical competence and facilitates sound judgement in the provision of quality healthcare. Furthermore, it enables critical risk-benefit appraisal to balance the principles of beneficence and non-maleficence to achieve a net benefit for patients.

## Non-maleficence

*Primum non nocere* (Latin for: first do no harm) Although healthcare practitioners are duty-bound to patients to optimise their health, they are fundamentally obligated to avoid and/or minimise harm. In the context of patient care, healthcare practitioners run the risk of causing harm where risk exceeds benefit. Regarding the use of ultrasound in primary care, for both diagnostic and therapeutic purposes, the risk must be critically appraised. Harm, as a medical error, can result from either acts of omission or commission,^10,11^ that is to say, from either not performing an ultrasound when it is necessary, or performing one when it is unnecessary (or incompetently, beyond one’s skillset).

Here, the ethics of consequentialism comes to play – regardless of what the doctor’s intentions are when using ultrasonography, the potentially harmful consequences include unnecessary bodily or mental harm to a patient, as well as damage to the medical profession (see *Apportionment of Damages Act 34 of 1956* below).

## Justice

South Africans have experienced generations of denial and violation of fundamental human rights. The post-apartheid constitution^12^ (Act No. 109 of 1996) stipulates that ‘all South Africans have the right to the progressive realisation of access to healthcare services’, regardless of their ability to pay.^13^ Furthermore, the constitution mandates the equitable distribution of the benefits of advancements in medical technology. It highlights the social value of research to target the burden of disease, be cost-effective, consider resource availability and focus on the health needs of vulnerable groups and communities.

## Legal considerations

There is no regulatory framework directly related to the use of PoCUS by non-radiologists and non-obstetric medical practitioners in South Africa. However, several Acts, as discussed below, guide the duties and responsibilities of practitioners on the use of new medical technologies.

### The *National Health Act* (Act no. 61 of 2003)^13^

Section 27(2) of the constitution holds that the government must employ reasonable legislative and facilitatory measures to achieve the progressive realisation of the right to access healthcare services (within its available resources). Simultaneously, Section 24(a) of the constitution holds that every citizen has the right to an environment that does not pose risk to their health or well-being.

### The *Health Professions Amendment Act* (Act No. 29 of 2007)^8^

Continuing professional development (CPD) is endorsed by the *Health Professions Amendment Act* (HPAA) as a method to update and maintain professional competence, to promote and protect public interest and to ensure optimal healthcare service to communities. CPD activities should align with the contemporaneous epidemiological transition model of South Africa and its disease burden.

Furthermore, the HPAA clearly delineates the process of acquiring additional qualifications and the mandate for professionals to practise within their scope of practice (similarly, unambiguously, stated in ethical rule 21 of the HPCSA Ethical Guidelines: ‘adequately trained through board-accredited measures, and sufficiently experienced’).^8^

### *Apportionment of Damages Act 34 of 1956* (ADA)^11^

Previously, public service employees were indemnified by the state under the principle of vicarious liability (*State Liability Act 20 of 1957*).^14^ However, with the exploding litigiousness of medical practice and growing global focus on human rights, state, defence, as well as plaintiff lawyers are shifting liability to the practitioner, particularly where the practitioner practises outside of his/her scope of practice as stipulated in the labour agreement. Increasingly, the concepts of delictual liability and contributory intent (*any* wrongful and blameworthy conduct which causes harm to a person) place greater responsibility on the practitioner and hold them liable for any damages incurred by any party.^11^

## Conclusion

Medicines and medical technologies are often viewed as private commodities.^2^ However, we must align our practice paradigm to that of the constitution: medical advancements are a public good, and South Africans have a right to the progressive realisation of access to these advancements in healthcare. A lack of training does not lower the expected standards of care. However, until such a time as the establishment of an accredited PoCUS programme by the CFPSA, the use of PoCUS falls outside of the scope of practice of family physicians and primary care practitioners, and the use thereof should be regarded as a delict.

It is arguable that both the evidence-base as well as the statutory framework should lead family physicians to embrace the use of PoCUS to supplement clinical care. Failing to do so could be regarded as an act of omission at both individual and system levels. Family physicians are obligated to lobby for legislative and regulatory frameworks that increase the universal availability and accessibility of PoCUS without discrimination and in an affordable fashion.^15^

Furthermore, family physicians are responsible for ensuring that the use of PoCUS is scientifically and medically appropriate, and of good quality. The use of PoCUS by the primary care specialist has theoretical underpinnings of preventive medicine, evidence-based medicine, sustainability and capacitation of the health system. A professional responsibility lies on the South African family physicians collectively to introduce rigorous training in the use of PoCUS by postgraduate students as well as oversight of accreditation and standard maintenance.

Future work should look to develop a robust situational assessment of the use of PoCUS. This could include a review of the literature on the uses of PoCUS in primary care, a quantitative assessment of common conditions presenting to South African primary care services that could guide curricular development. Other potential areas could include an audit of current views and practices amongst primary care practitioners, and an expert consensus model (e.g. modified Delphi) to inform curriculum development. Pilot projects, perhaps conducted within the custodianship of established registrar training programmes, should be closely monitored and evaluated for patient safety and responsible PoCUS uptake with iterative refinement of curricula.

## References

[1] American College of Emergency Physicians. Emergency ultrasound guidelines. Ann Emerg Med. 2009;53(4):550–570. 10.1016/j.annemergmed.2008.12.013 19303521

[2] Becker DM, Tafoya CA, Becker SL, Kruger GH, Tafoya MJ, Becker TK. The use of portable ultrasound devices in low- and middle-income countries: A systematic review of the literature. Trop Med Int Health. 2016;21(3):294–311. 10.1111/tmi.12657 26683523

[3] Hall JWW, Holman H, Bornemann P, et al. Point of care ultrasound in family medicine residency programs: A CERA Study. Fam Med. 2015;47(9):706–711. 26473563

[4] Walton J. Ultrasound education and training in the new NHS – Setting standards – A personal viewpoint: BMUS Bull [homepage on the Internet]. 2016 [cited 2020 May 31]; Available from: https://0-journals-sagepub-com.innopac.wits.ac.za/doi/pdf/10.1177/1742271X0100900106

[5] Miller DL, Abo A, Abramowicz JS, et al. Diagnostic ultrasound safety review for point-of-care ultrasound practitioners. J Ultrasound Med. 2020;39(6):1069–1084. 10.1002/jum.15202 31868252

[6] Lamprecht H, Stander M, Van Hoving N. Emergency point-of-care ultrasound applications. Contin Med Educ. 2012;30(11):416–419.

[7] The Colleges of Medicine of South Africa. Constitution of the College of Family Physicians [homepage on the Internet]. [cited 2021 Aug 01]. Available from: https://www.cmsa.co.za/view_college.aspx?collegeid=6

[8] The Health Professions Council of South Africa. Professional conduct & ethics - HPCSA [homepage on the Internet]. [cited 2021 Aug 10]. Available from: https://isystems.hpcsa.co.za/Conduct/Ethics

[9] Moodley KE. Ethics, law and human rights: A South African perspective. Contin. Med. Educ. 2008 Jan 10;24(1):5.

[10] SAFLII search. SAFLII [homepage on the Internet]. [cited 2020 Feb 17]. Available from: http://www.saflii.org/cgi-bin/sinosrch-adw.cgi?query=ultrasound&submit=Search

[11] Ahmed R. Contributory intent as a defence excluding delictual liability. S Afr Law J. 2014;131(1):88–108.

[12] The South African Government. Constitution of the Republic of South Africa, 1996 [home page on the Internet]. [cited 2021 Mar 25]. Available from: https://www.gov.za/documents/constitution-republic-south-africa-1996

[13] The South African Government. *National Health Act* 61 of 2003 [home page on the Internet]. [cited 2021 May 22]. Available from: https://www.gov.za/documents/national-health-act

[14] The South African Government. *State Liability Act* 20 of 1957 [home page on the Internet]. [cited 2021 Jun 2]. Available from: https://www.gov.za/documents/state-liability-act-5-apr-1957-0000

[15] World Health Organisation. Imaging modalities [homepage on the Internet]. WHO. [cited 2020 Feb 21]. Available from: http://www.who.int/diagnostic_imaging/imaging_modalities/en/

